# Analysis of the Distribution Pattern of *Phenacoccus manihoti* in China under Climate Change Based on the Biomod2 Model

**DOI:** 10.3390/biology13070538

**Published:** 2024-07-17

**Authors:** Yumeng Huang, Tong Li, Weijia Chen, Yuan Zhang, Yanling Xu, Tengda Guo, Shuping Wang, Jingyuan Liu, Yujia Qin

**Affiliations:** 1 Key Laboratory of Surveillance and Management for Plant Quarantine Pests, Ministry of Agriculture and Rural Affairs, College of Plant Protection, China Agricultural University, Beijing 100193, China; hym921604@163.com (Y.H.); islitong@163.com (T.L.); zy18335462253@163.com (Y.Z.); xyl0402@163.com (Y.X.); guotd7911@163.com (T.G.); 2 Technical Center for Animal, Plant and Food Inspection and Quarantine of Shanghai Customs, Shanghai 200135, China; weijia_0801@163.com (W.C.); wangshuping_2014@126.com (S.W.)

**Keywords:** *Phenacoccus manihoti*, climate change, Biomod2, geographical potential distribution, distribution center, ecological niche

## Abstract

**Simple Summary:**

The cassava mealybug (*Phenacoccus manihoti*) represents a significant quarantine pest affecting cassavas. This study seeks to elucidate the patterns of the suitable habitat and distribution centers of cassava mealybugs in China, as well as their dynamic ecological niche in invaded areas under the influence of climate change. Climate factors have primarily concentrated suitable habitats for cassava mealybugs in the southern regions of China, with minimal observed changes and a slight northward expansion. The dynamic ecological niche is also projected to expand slightly. Based on these predictions, relevant quarantine agencies can implement measures to prevent the introduction and spread of cassava mealybugs.

**Abstract:**

The changing global climate has significantly impacted the spread of plant pests. The cassava mealybug (*Phenacoccus manihoti*) is among the most dangerous quarantine pests affecting cassavas worldwide, causing substantial losses in agricultural production and food security across several regions. Although China is currently free of the cassava mealybug, its proximity to affected countries and extensive trade with these regions necessitate a detailed understanding of the pest’s distribution pattern and dynamic ecological niche changes. Using the Biomod2 model, we selected two historical climate scenarios and two future climate scenarios (SSP1-2.6 and SSP5-8.5) to investigate the distribution patterns, potential habitats, distribution centers, and dynamic ecological niches of cassava mealybugs in China. Key environmental variables influencing the distribution were identified, including bio4, bio8, bio12, bio18, and bio19. The potential habitat of cassava mealybugs is mainly located in several provinces in southern China. In the future, the suitable habitat is projected to expand slightly under the influence of climate change, maintaining the overall trend, but the distribution center of suitable areas will shift northward. Dynamic ecological niche prediction results indicate the potential for further expansion; however, the ecological niches may be unequal and dissimilar in the invaded areas. The predictions could serve as a valuable reference for early warning systems and management strategies to control the introduction of cassava mealybugs.

## 1. Introduction

The invasion of alien species presents a significant global threat. The spread of these species is driven by anthropogenic factors such as international travel and trade, as well as natural factors. Insects are the most prevalent invasive species, substantially contributing to ecological disruption. Global climate warming is a major natural factor contributing to increased invasions of harmful pests [1,2,3]. Climate change influences pest distribution, posing significant threats to crops, as discussed in some literature [4,5,6]. Due to global warming, the spread of invasive crop pests is projected to increase, impacting a wider range of cultivated crops [6,7]. The latest phase of the Coupled Model Intercomparison Project (CMIP6) offers significant advancements over CMIP5. These advancements include the transition from simple ocean–atmosphere-coupled models to sophisticated multi-layer models that integrate the atmosphere, land surface, ocean, and sea ice. Additionally, CMIP6 incorporates a comprehensive array of human-activity-related factors such as carbon cycles, vegetation cover, population dynamics, economic growth, education, urbanization, and technological progress, all of which interact with the climate system’s physical, biological, and chemical processes [8]. Among the four Shared Socioeconomic Pathways (SSPs) provided by CMIP6 [9], SSP1-2.6 represents a lower forcing scenario, where radiative forcing is predicted to reach 2.6 W/m^2^ by 2100, potentially keeping the temperature increase below 2.0 °C relative to pre-industrial levels. SSP2-4.5 and SSP3-7.0 correspond with medium forcing scenarios, with radiative forcing reaching 4.5 W/m^2^ and 7.0 W/m^2^, respectively, by 2100. SSP5-8.5 represents the highest forcing scenario, with radiative forcing reaching 8.5 W/m^2^ by 2100 [10].

The cassava mealybug (CMB), *Phenacoccus manihoti* Matile-Ferrero (Hemiptera: Pseudococcidae), poses a significant threat to cassava production worldwide and is recognized as one of the most critical quarantine pests of cassava crops [11,12,13]. It is an oligophagous pest that can feed on and damage a variety of economic crops and ornamental plants, including cassava, rubber cassava, sweet potato, soybean, citrus, tomato, pepper, and poinsettia. However, it prefers to proliferate on cassava and rubber cassava. Other hosts merely serve as temporary feeding sites during population dispersal and do not result in an economic impact or the production of offspring on these plants [14]. Originating from South America [15], this mealybug was introduced into Africa in the early 1970s and subsequently into Asia (Thailand) in 2009, emerging as the most destructive pest of cassava globally [16,17]. This insect reproduces parthenogenetically, resulting in exclusively female offspring. Consequently, a single nymph or adult can initiate an infestation. Under optimal conditions, an adult female can lay between 200 and 600 eggs within ovisacs [18,19]. The pest feeds on plant tissues as both nymphs and adults, causing apical buds and leaves to curl and develop poorly, reducing the internode length and weakening stems and roots. This frequently results in yield reductions exceeding 50%, with severe infestations potentially causing total crop loss and plant death [20,21]. Additionally, the pest can induce sooty mold, further exacerbating plant damage. Currently, the cassava mealybug is widespread and severely affects major cassava-producing regions worldwide [12,13]. In Africa, it spreads at an alarming rate of 150 km per year compared with fewer than 30 km per year for other invasive hemipterans [22]. Control of the cassava mealybug primarily relies on chemical and biological methods [15]. However, the number of registered pesticides for this pest is very limited, and biological control is often ineffective due to the low diversity of natural enemies in cassava-growing areas, leading to unstable predator populations. Therefore, finding new, effective, and environmentally friendly strategies to control the cassava mealybug has become a critical issue for the development of the cassava industry, both domestically and internationally [23].

Originally native to South America, the cassava mealybug (CMB) has now spread across multiple countries and regions in Asia, Africa, and South America [24]. Introduced to the African continent in the early 1970s, it has become naturalized throughout sub-Saharan Africa. First detected in Asia in 2008 in Thailand, it quickly spread across cassava-growing regions and into neighboring countries, including Indonesia, raising significant concerns about its potential spread to other countries [21,25].

A pest risk analysis (PRA) involves evaluating biological, scientific, and economic evidence to determine whether an organism is a pest, if it should be regulated, and the strength of any phytosanitary measures required. It is a crucial component of plant quarantine [26,27]. Species distribution models (SDMs) can predict species distribution areas and are widely used in fields such as endangered species conservation [28], conservation area planning [29], invasive species control [30], and predicting the impact of climate change on species distributions [31]. Currently, there are numerous distributional prediction models, with various principles, assumptions, algorithms, scopes of application, and prediction performances. Thuiller [32] suggests that each model has its own advantages and limitations due to the different principles and algorithms, and model performance is not stable with a change in input data. Rather than relying on a single model, it is preferable to establish a model ensemble, integrating results from multiple models to enhance prediction accuracy by using a comprehensive result as the output of the ensemble. Biomod2, an R package used to predict the potential geographical distribution of organisms, provides a range of tools for model selection, model fusion, and model evaluation that can be used to predict the spatial distribution of biodiversity under different environmental variables [33]. Similar to the maximum entropy model (MaxEnt) and other biological diversity models that correlate species distribution data with environmental factors to build models, Biomod2 can integrate generalized linear models (GLMs), random forests (RFs), MaxEnt, and artificial neural networks (ANNs), so it has the advantage of being more accurate and reliable than a single model [33,34,35]. Biomod2 is widely used to predict species distributions, assess the impact of climate change on biodiversity, and explore the relationship between biodiversity and environmental factors [36,37]. Numerous studies on potential geographic distributions based on the Biomod2 model have been conducted, both domestically and internationally. Liu et al. [38] used Biomod2 to predict the potential distribution of *Teinopalpus aureus* Mell. De Oliveira et al. [39] modeled the ecological niche and evaluated the remaining suitable habitat areas for the occurrence of five potentially invasive species of freshwater decapods in South America. Shabani et al. [40] used Biomod2 to investigate changes in the invasion risk of the Dubas bug under current and future climate change scenarios. In this study, we used Biomod2 integrated with an optimal combination model and ArcGIS to predict the shifts in potential suitable habitats, distribution centroids, and the distribution pattern of *P. manihoti*, aiming to provide a scientific basis for effective early warning management under climate change.

## 2. Materials and Methods

### 2.1. Distribution Records of Phenacoccus manihoti

**Figure 1:**
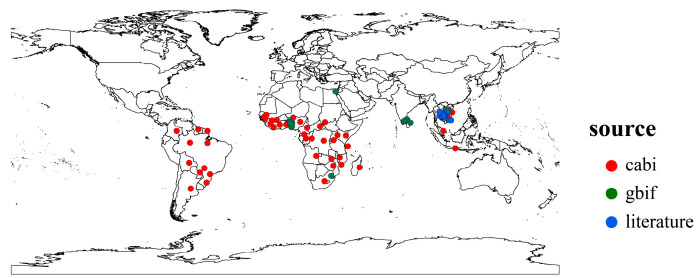
Global distribution points of *Phenacoccus manihoti*.

We collected distribution records of *P. manihoti* from the Center for Agriculture and Bioscience International (CABI) [24], the Global Biodiversity Information Facility (GBIF) [41], and related literature [13,42]. Initially, we removed specimen coordinates or incorrect distribution data, then used the spThin package (version 0.2.0) to retain only one distribution point per 5 min × 5 min raster to avoid a spatial sample bias [43]. Ultimately, we obtained 102 occurrence records of *P. manihoti* for the model setting (Figure 1). We imported the distribution records into the model using the BIOMOD_FormatingData function in the Biomod2 package (version 4.2-4), controlling the generation of pseudo-absences (PA) using the PA.nb.rep, PA.nb.absences, and PA.strategy parameters. In this study, we generated pseudo-absence points by randomly generating 10 instances and repeating the generation 3 times.

### 2.2. Environmental Variables

We obtained elevation (ele) data as well as historical (1970–2000) and future (2040–2060) (the 2050s) data for 19 bioclimatic variables (Table A1) in Worldclim version 2.1 (https://www.worldclim.org, accessed on 2 May 2024), with a spatial resolution of 5 arc_min. For future environmental data, we selected two different Shared Socioeconomic Pathways (SSP1-2.6 and SSP5-8.5) for 2050 in the BCC-CSM2-MR model. To ensure a better response of the variables used in the modelling, a pre-run of the MaxEnt model was constructed in R using the terra package and the SDMtune package to pre-screen the 20 climatic variables, excluding those with a contribution of fewer than 5 [44]. Correlation analyses were then performed to exclude variables with a strong covariance (|r| > 0.8) to avoid excessive correlations between variables [45].

### 2.3. Model Settings and Assessment

A single model was modelled for each species using the BIOMOD_ModelingOptions and BIOMOD_Modeling functions (This package was developed by Wilfried Thuiller and his research team at the Laboratoire d’Écologie Alpine (LECA) and Centre National de la Recherche Scientifique (CNRS) in Grenoble, France.), resulting in the selection of 11 single models. These were the generalized linear model (GLM) [46], generalized additive model (GAM) [47], generalized boosting model (GBM) [48] (pp. 103–105), classification tree analysis (CTA) [49], artificial neural network (ANN) [50], surface range envelop (SRE) [51], flexible discriminant analysis (FDA) [52], multiple adaptive regression splines (MARS) [53] (pp.1–15), random forest (RF) [54], eXtreme Gradient Boosting (XGBOOST) [55], and maximum entropy model (MaxEnt) [56]. The parameters of the MaxEnt model were tuned using the ENMeval package. The model was controlled by tuning the parameters CV.nb.rep, prevalence, and metric.eval. The model was run 10 times, with the training-set data proportion set to 75% [57]. The prevalence was set to 0.5, meaning the sum of the weights of the distributed points equaled the sum of the weights of the pseudo-absence points. The true skill statistic (TSS) measures the net prediction success rate of a model using distribution points and pseudo-distribution points, with values ranging from −1 to 1. The closer the TSS is to 1, the higher the model’s prediction accuracy; the closer it is to −1, the more the model tends to be stochastic [58,59]. The receiver operating characteristic (ROC) curve is chosen as a criterion to evaluate the model performance [58]. Models utilized for integrated model building are required to have ROC values higher than 0.8 and TSS values exceeding 0.7 [55,60].The BIOMOD_EnsembleModelling function utilizes the em.algo parameter to choose from six methods for integrated modelling. The options include EMmean (based on the probability mean of the selected model), EMmedian (based on the median probability of the selected model), EMcv (based on the probability coefficient of variation in the selected model, i.e., SD/mean), EMci (based on the confidence interval around the probability mean of the selected model), EMca (based on the binary voting of the selected model), and EMwmean (based on the evaluation score), with the selected model’s probabilities weighted by the evaluation scores obtained at the time of model construction. Among these, EMmedian is less influenced by extreme anomalies compared with EMmean, yet it demands greater computational resources and memory. EMmean assesses uncertainty rather than the likelihood of species occurrence. In EMwmean, the better the previous model evaluation, the greater the weight in the later integration.

### 2.4. Analysis and Mapping

The results of the BIOMOD_EnsembleForecasting function were evaluated for the TSS and ROC scores, and the appropriate integrated model was selected. The Jenks natural break classification (NBC) method was used to classify suitable areas [61,62]. After developing the integrated model, the BIOMOD_EnsembleForecasting function projected the integrated model onto the environmental variables for the potential geographic distribution prediction and the distribution maps were normalized using ArcGIS 10.2. According to the results from Biomod2, the thresholds for unsuitable, low-suitable, medium-suitable, and high-suitable areas for cassava mealybugs were 0–91.42829, 91.42829–232.03453, 232.03453–402.96921 and 402.96291-1000, respectively. The data from different climatic conditions were reclassified and assigned to determine future changes in suitable habitats for the four climatic scenarios compared with historical conditions using the raster calculator. Additionally, the SDM toolbox v 2.5 plug-in was used to calculate the center of cassava mealybug populations for each period and to plot the trajectory of the center of mass migration.

### 2.5. Ecological Niche Analysis

We assessed the degree of geographical matching between invasive and native habitats by overlapping the corresponding binary maps obtained for each temporal scenario. The environmental principal component analysis (PCA-env) in the R package ecospat (version 3.5.1) [63,64] was used for this study. We then employed niche equivalency and background similarity tests to evaluate the observed similarities between each pairwise comparison [64,65]. Schoener’s D metric determined the extent of the ecological niche overlap, with index ranges from 0 (no niche overlap) to 1 (complete niche overlap), where larger values indicated a higher overlap rate of ecological niches. To understand the importance of niche overlap in geographic areas, we conducted niche equivalency and similarity tests [64]. The ecological niche equivalency test was conducted by comparing the ecological niche overlap (D) values for current and future climate scenarios with the overlap of the null distribution. The niche similarity test assessed if the ecotopes of the two entities being compared were more similar (or different) than expected, considering the surrounding environmental conditions across the geographic area [64,66]. We randomly repeated the tests 1000 times, and the null hypotheses of niche equivalence and similarity were accepted if the observed niche values (D) were significantly higher than the overlap value from the null distribution (*p* < 0.05) [64,67].

## 3. Results

### 3.1. Model Validation

**Figure 2:**
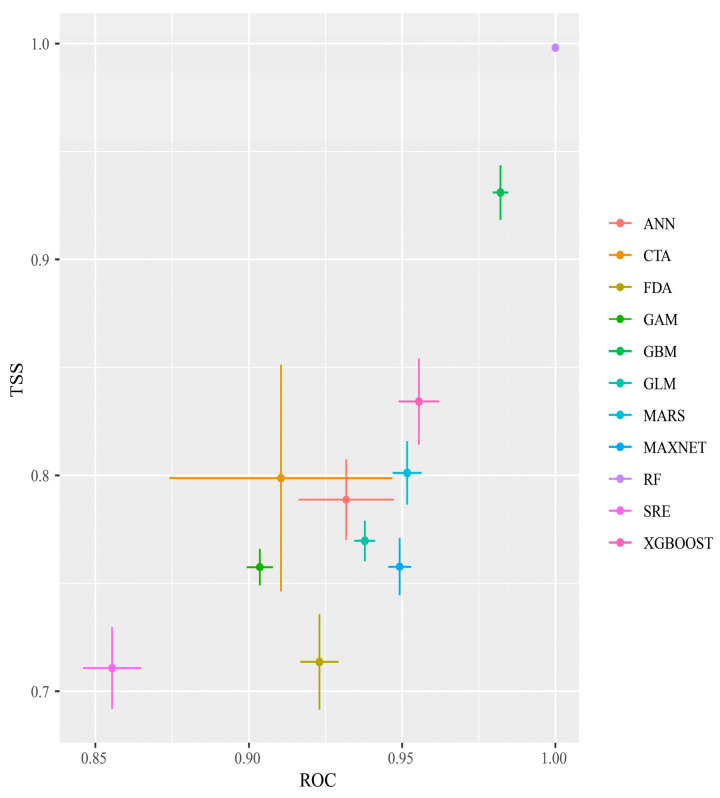
TSS and ROC evaluations of each single model of *Phenacoccus manihoti*.

After 10 repeated runs of the single and combined models, and based on the TSS and ROC evaluations, RF had the highest TSS and ROC values, and SRE had the lowest TSS and ROC values, although its TSS was still >0.7 and its AUC was >0.85. All 11 models met the screening criteria (Figure 2) and were, therefore, used to construct the integrated model.

For the integrated modeling, this study chose the EMwmean method, based on previous research [68]. The ROC value of this integration approach was 0.970 and the TSS value was 0.807, indicating high predictive accuracy suitable for the outcome prediction.

### 3.2. Environmental Variables

Using the SDMtune package, the key environmental factors influencing the distribution of *P. manihoti* were identified as bio4 (temperature seasonality), bio8 (mean temperature of the wettest quarter), bio12 (annual precipitation), bio18 (precipitation of the warmest quarter), and bio19 (precipitation of the coldest quarter). These five factors were, therefore, included in the final model.

**Figure 3:**
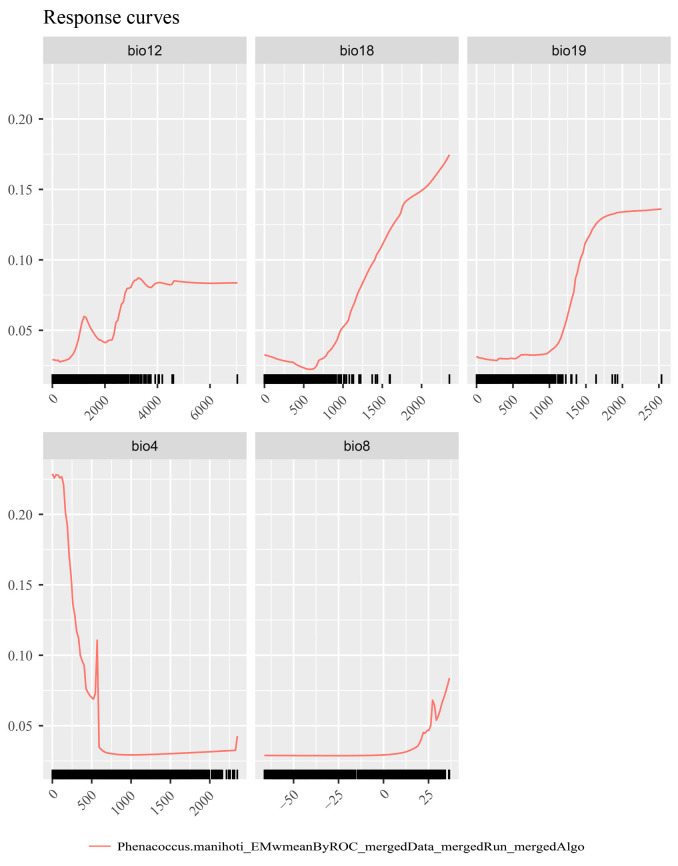
Response curves of *Phenacoccus manihoti*.

Environmental factor response curves reflect the effect of environmental variables on habitat suitability [69]. According to the response curve of cassava mealybugs (Figure 3), bio4 (temperature seasonality) varied greatly within a small range of values, bio8 (mean temperature of the wettest quarter) varied significantly at 25 °C, bio12 (annual precipitation) varied significantly around 4000 mm, bio18 (precipitation of the warmest quarter) showed high suitability at about 500 mm, and bio19 (precipitation of the coldest quarter) showed significant changes and higher suitability at about 1000 mm. The response curves indicated that cassava mealybugs prefer warm and stable temperature conditions.

### 3.3. Potential Geographical Distribution in China

**Figure 4:**
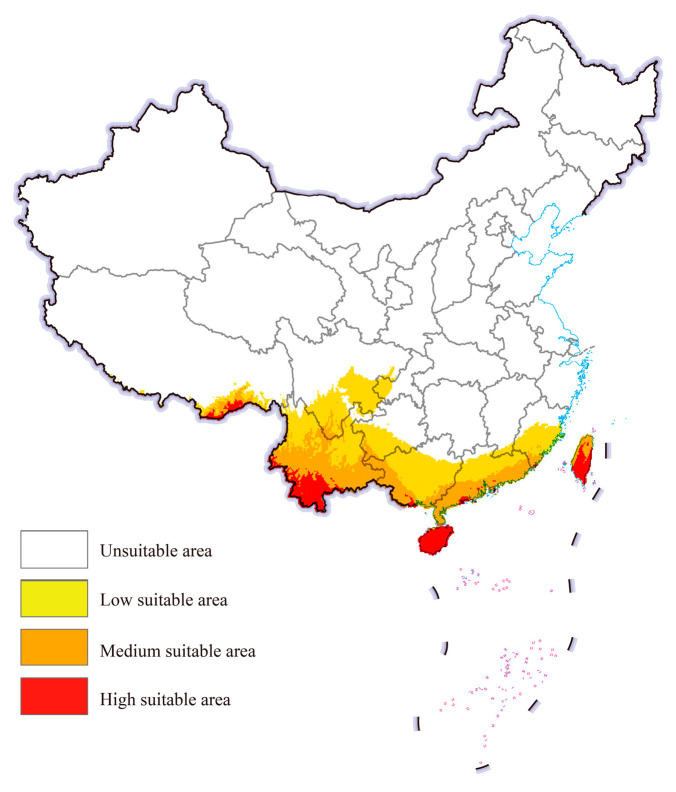
Potential geographic distribution of *Phenacoccus manihoti* in China under a historical climatic scenario.

The potential geographical distribution of cassava mealybugs worldwide under historical climatic conditions was predicted by the integrated model using EMwmean (Figure 4). In China, cassava mealybugs were generally found in fewer areas. High-suitable areas mainly existed in parts of southeastern Tibet and southern Yunnan, as well as in Taiwan, most of Hainan province, and scattered areas in Guangdong and Guangxi. The suitable area expanded inland from high-suitable areas and could be found in provinces such as Yunnan, Guizhou, and Fujian.

**Figure 5:**
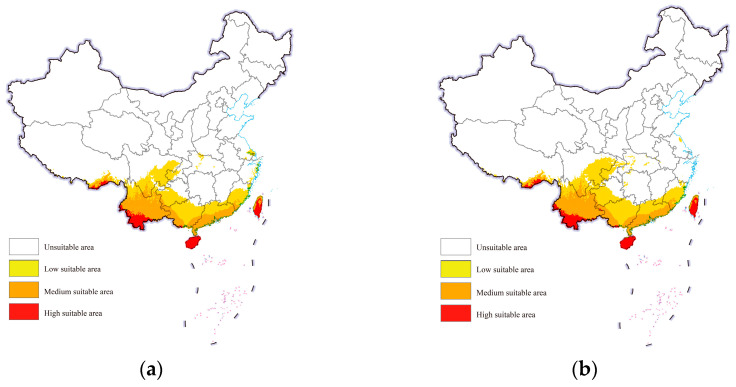
Potential geographic distribution of *Phenacoccus manihoti* in the world under future climatic scenarios: (**a**) SSP1-2.6; (**b**) SSP5-8.5.

Under both future scenarios, the suitable areas for cassava mealybugs within China were still mainly concentrated in the aforementioned provinces. However, a small number of low-suitable areas were also observed in Chongqing, Hubei, Jiangsu, and Zhejiang from the projections (Figure 5). The overall change in the area of each suitability class was not significant. Under future climate scenarios, the low-suitable area could reach further than 700,000 square kilometers and the medium-suitable area could increase from more than 400,000 square kilometers to more than 500,000 square kilometers (Table 1).

### 3.4. Shifts in the Distribution Centroid and Distribution Pattern

**Figure 6:**
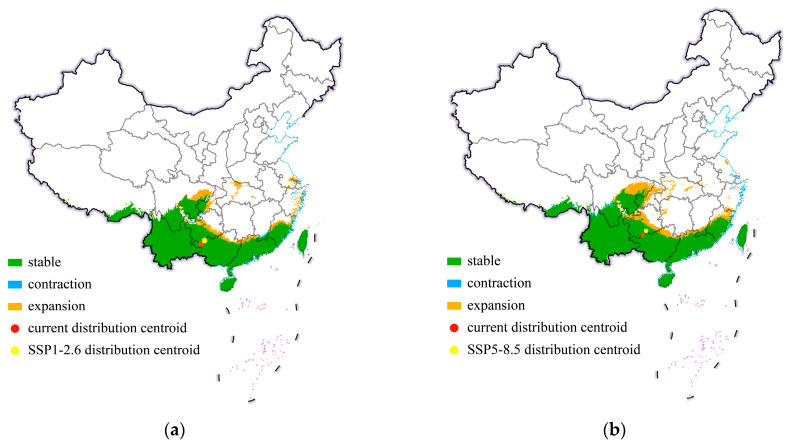
Shifts in potential suitable habitats of *Phenacoccus manihoti* in China under different climate scenarios: (**a**) SSP1-2.6; (**b**) SSP5-8.5.

In this study, we projected the shift in the center point of distribution of cassava mealybugs to reveal the invasion trend of this species in China. The shifts in the potential suitable habitats of *P. manihoti* in China under historical conditions and different future climate scenarios in 2050 are shown in Figure 6. Within China, there was no significant change in its suitable area. Specifically, under the SSP1-2.6 climate scenario, the suitable habitats of the cassava mealybug mainly showed a small expansion trend, but the expansion was limited to the vicinity of the original suitable area. Additionally, there was a small-scale contraction trend in the habitable area in southern Jiangxi. Figure 6 shows the centroid shift trend of cassava mealybugs in China under two scenarios (SSP1-2.6 and SSP5-8.5) from the current climate to 2050. In both climate scenarios, the distribution center of cassava mealybugs showed a northward-spreading trend, but both were concentrated in Guizhou, with a greater northward shift in the SSP5-8.5 climate scenario. The small shift in the distribution center also indicated that the change in the suitable area was not significant under different future climate scenarios.

### 3.5. Ecological Niche of Phenacoccus manihoti

**Figure 7:**
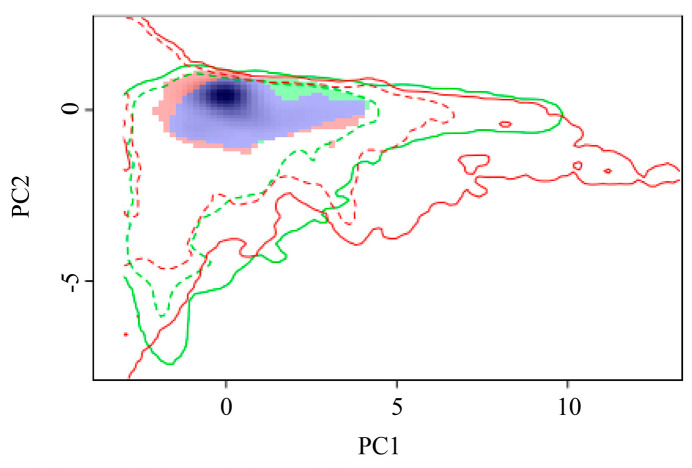
Global variation in the ecological niche of *Phenacoccus manihoti*: red and green blocks indicate expansion and unfilled areas, respectively; purple blocks indicate overlapping niches.

The geographical ranges of *P. manihoti* can be determined, in part, by its fundamental niche, i.e., the environmental conditions under which the cassava mealybug can survive. The variation in the ecological niche of *P. manihoti* was verified by comparing the differences in the ecological space between native and invaded countries (Figure 7). To assess the indicators of ecological niche dynamics, a set of environments was available for both the current and future scales; if the environment was only occupied in the future scale, this represented ecological niche expansion. Similarly, environments were considered to exhibit ecological niche stability if they were occupied in both the current and future scales, and ecological niche vacancies if they were used in the current scale and were available, but not yet utilized, in the future scale [70]. Niche analyses contrasting current and future climatic conditions showed that the ecological niche overlap index (D) was 0.56, revealing that the cassava mealybug had currently occupied all of its niches and was likely to expand on a small scale. The niche similarity (*p* = 0.15984 > 0.05) and equivalence (*p* = 0.1938 > 0.05) tests for the null hypothesis were not rejected, suggesting that the ecological niches of the cassava mealybug were not statistically equivalent and the entities to be compared were similar. We hypothesized that the ecological niche occupied by the cassava mealybug changed to some extent during its invasion.

## 4. Discussion

The cassava mealybug is widely distributed worldwide and has caused significant economic losses, particularly in regions where cassava is extensively cultivated such as Africa and India [16,71]. Although it has not yet been introduced into China, it has attracted national attention due to its presence in neighboring countries such as Thailand, Laos, and Myanmar. In 2011, it was included in the Catalogue of Quarantine Pests for Import Plants of the People’s Republic of China. This study is the first to use the Biomod2 model to investigate the potential geographical distribution, patterns, and dynamic ecological niches of the cassava mealybug in China, with the aim of providing a theoretical basis for their monitoring in the country.

### 4.1. Optimization and Selection of the Biomod2 Model

Integrated models have the advantage of being more accurate and reliable than single models [32]. Different single models have their own characteristics; for example, the MaxEnt model is specifically designed to simulate the distribution of species with only available data and is suitable for situations with a greater amount of missing data [72], while SRE is suitable for situations with a greater number of variables and can adapt to reduce the complexity of the model [73]. However, most studies using integrated models only employ the default parameters of each single model [74]. In this study, the MaxEnt model in the model combination was optimized for parameter tuning and then included in the integrated model construction. Further model optimization and parameter tuning can be carried out in future studies. Judging by the ROC and TSS, all the models in this study had better results when run separately. When integrating the models, the ROC value of this model integration approach was 0.970 and the TSS value was 0.807, indicating that our prediction results were better. Biomod2 performed well in terms of the accuracy of the model prediction results, but it also had some drawbacks such as requiring more data preprocessing steps, a longer runtime, and larger storage-space requirements at runtime, making it less simple and easy to understand compared with a single MaxEnt [34]. Overall, Biomod2 combines a variety of models to provide better predictions with fewer input distributions [75,76,77].

### 4.2. Significant Environmental Variables

We identified five key environmental variables affecting cassava mealybugs using the SDMtune package. These were bio4 (temperature seasonality), bio8 (mean temperature of the wettest quarter), bio12 (annual precipitation), bio18 (precipitation of the warmest quarter), and bio19 (precipitation of the coldest quarter). Previous studies have shown that the main factors affecting the survival and population growth of cassava mealybugs are temperature and rainfall, which align with our findings [13]. It was found that the optimal temperature for development was 27 °C, with a significant number of deaths occurring when the temperature was lower than 15 °C or higher than 33 °C [13,19]. The response curves of the environmental variables also showed a better response for bio8 above 25 °C, while the best response value of bio4 was smaller, indicating less tolerance for temperature changes such as completing physiological activities like overwintering. The cassava mealybug is mainly found in countries near the equator where temperatures do not vary much, according to existing distribution sites. Additionally, its growth is constrained by precipitation: dry areas, years, and seasons favor outbreaks [13]. The response curves for bio12, bio18, and bio19 also showed that cassava mealybugs depend on a moderate amount of precipitation, which is in line with their own physiological characteristics. However, our selection of relevant bioclimatic variables considered only 19 climatic and altitudinal variables and did not account for anthropogenic impacts, soil environment, and the pest’s physiological data. The potential habitat areas predicted by some bioclimatic variables may not be suitable for the species to live in reality, which is a limitation of the classical bioclimatic variables used in this study.

### 4.3. Distribution Pattern and Ecological Niches

Many models and methods have been used to predict the potential geographical distribution of cassava mealybugs. Soroush et al. [13] reported recent distribution records and estimated the climatic suitability for their regional spread using a CLIMEX distribution model. Chen et al. [78] analyzed the potential geographical distribution of the cassava mealybug in China, using three different ecological modeling methods (Maxent, ENFA, and Mahalanobis typicalis) to predict its potential distribution. Tania et al. [71] used CLIMEX to study its global distribution and its threat to food security for the poor. Zhou et al. [14] used the GARP ecological niche model and related environmental data combined with the interpolation and overlay functions of ArcGIS to predict that the potential habitat of cassava mealybugs in China would mainly be concentrated in the area south of 25° N. The prediction results of various scholars have varied slightly due to different factors such as the models and variables used, but the overall trends were similar. In this study, the potential geographic distribution of cassava mealybugs was studied using ensembled models. The results showed that this species would mainly be concentrated in the southern region of China in provinces near Laos, Myanmar, and other countries, as well as in Taiwan and Yunnan, where the temperature is higher. With climate change, the expansion of the cassava mealybug’s potential habitat was not obvious, and we speculated that temperature would limit its further expansion under natural conditions. Within China, the range of its habitat was not wide; moreover, cassava cultivation within China is mainly concentrated in some cities in the south [79]. In this study, the climate scenarios of SSP1-2.6 and SSP5-8.5 showed different degrees of expansion of the habitats and the distribution center of the suitable areas shifted northwards to varying degrees, suggesting that climate change would affect the distribution pattern of cassava mealybugs. It is crucial to prevent the northward invasion of mealybugs. According to the results of the dynamic ecological niche analysis, cassava mealybugs could invade from their origin to other places, but their ecological niche would change to some extent. We deduced that this may be due to the species’ physiological characteristics, host distribution, or the existence of interspecific competition and these aspects should be focused on in subsequent studies. Therefore, it is important to monitor the dynamics of cassava mealybug occurrences in new areas in a timely manner and to enhance its small-scale prediction.

### 4.4. Management and Monitoring

The cassava mealybug is currently absent from China, but it is a quarantine pest of concern in China. The first instar of this insect is very active and can crawl from infected plants to neighboring healthy plants, and can also spread with wind, animals, or equipment. Long-distance transmission is mainly through the transport of infected host plants and their products. This was the case for the cassava mealybug’s spread from South America to Africa and then to Thailand [80] (pp. 209–235). Therefore, as a neighboring country to Thailand, Laos, Myanmar, and other countries with a wide distribution of cassava mealybugs, and one that imports large quantities of cassava products from many neighboring countries [20], China should strengthen border quarantine measures and strictly prevent and control the importation of cassava mealybugs.

## 5. Conclusions

In this study, we used the Biomod2 model to identify the key environmental variables affecting the distribution of cassava mealybugs by selecting historical scenarios and two future climatic scenarios, SSP1-2.6 and SSP5-8.5. We studied the distribution pattern of the cassava mealybugs’ potential habitat, distribution centers, and dynamic ecological niches in invaded areas within China. Our findings indicated that bio4 (temperature seasonality), bio8 (mean temperature of the wettest quarter), bio12 (annual precipitation), bio18 (precipitation of the warmest quarter), and bio19 (precipitation of the coldest quarter) had a non-negligible effect on the distribution of cassava mealybugs. Under the historical scenario, cassava mealybugs were mainly distributed in a few provinces in southern China, with highly suitable areas concentrated in Yunnan, Taiwan, and Hainan. Under the two future climatic scenarios, SSP1-2.6 and SSP5-8.5, the distribution of suitable areas for cassava mealybugs slightly expanded, but the overall trend remained the same. The center of distribution for suitable areas moved northwards, but remained concentrated in Guizhou. Dynamic ecological niche projections showed further potential for expansion, but the ecological niches in invaded areas was statistically not equivalent in ecological terms and did not indicate that the ecological niches were more similar than expected. This study is the first to use the Biomod2 model to investigate the potential habitats, habitability patterns, and dynamic ecological niches of the cassava mealybug in China, providing theoretical support for their monitoring in the country.

## Figures and Tables

**Table 1:** Potential suitable habitats area of *Phenacoccus manihoti* under different climate scenarios in China.

Climate Scenario	Area (×10^4^ km^2^)		
	Low-Suitable Area	Medium-Suitable Area	High-Suitable Area
Historical climatic scenario	645,489.1268	411,300.4540	186,008.8396
SSP1-2.6 (2050)	719,153.7544	510,826.8700	178,921.3524
SSP5-8.5 (2050)	766,730.3972	506,906.1324	192,417.7376

## Data Availability

The original contributions presented in the study are included in the article, further inquiries can be directed to the corresponding authors.

## Appendix A

**Table A1:** Environmental variables (bold indicates variables used for modeling).

Types of Variables	Environmental Variables	Code
Climatic factors	Annual mean temperature	bio1
	Mean diurnal range	bio2
	Isothermality	bio3
	Temperature seasonality	bio4
	Max temperature of warmest month	bio5
	Min temperature of coldest month	bio6
	Temperature annual range	bio7
	Mean temperature of wettest quarter	bio8
	Mean temperature of driest quarter	bio9
	Mean temperature of warmest quarter	bio10
	Mean temperature of coldest quarter	bio11
	Annual precipitation	bio12
	Precipitation of wettest month	bio13
	Precipitation of driest month	bio14
	Precipitation seasonality	bio15
	Precipitation of wettest quarter	bio16
	Precipitation of driest quarter	bio17
	Precipitation of warmest quarter	bio18
	Precipitation of coldest quarter	bio19
Terrain factors	Elevation	elev
